# A cross sectional observational study of research activity of allied health teams: is there a link with self-reported success, motivators and barriers to undertaking research?

**DOI:** 10.1186/s12913-017-1996-7

**Published:** 2017-02-06

**Authors:** Rachel J. Wenke, Sharon Mickan, Leanne Bisset

**Affiliations:** 1 Gold Coast Health, 1 Hospital Boulevard, Southport, Qld 4215 Australia; 2 School of Allied Health Sciences, Menzies Health Institute Queensland, Griffith University, Southport, Qld 4215 Australia; 3 Physiotherapy, School of Allied Health Sciences, Menzies Health Institute Queensland, Griffith University, Southport, Qld 4215 Australia

**Keywords:** Allied health professionals, Research capacity building, Motivation, Research activity

## Abstract

**Background:**

Team-based approaches to research capacity building (RCB) may be an efficient means to promote allied health research participation and activity. In order to tailor such interventions, a clearer understanding of current patterns of research participation within allied health teams is needed. Different self-report measures exist which evaluate a team’s research capacity and participation, as well as associated barriers and motivators. However, it remains unclear how such measures are associated with a team’s actual research activity (e.g., journal publications, funding received). In response, this observational study aimed to identify the research activity, self-reported success, and motivations and barriers to undertaking research of eight allied health professional (AHP) teams and to explore whether any relationships exist between the self-reported measures and actual research activity within each team.

**Methods:**

A total of 95 AHPs from eight teams completed the research capacity and culture survey to evaluate team success, barriers and motivators to undertaking research, and an audit of research activity from January 2013 to August 2014 was undertaken within each team. Kendell’s correlation coefficients were used to determine the association between research activity (i.e., number of journal publications, ethically approved projects and funding received) and the self-reported measures.

**Results:**

Seven out of eight teams rated their teams as having average success in research and demonstrated some form of research activity including at least two ethically approved projects. Research activity varied between teams, with funding received ranging from $0 to over $100,000, and half the teams not producing any journal publications. Team motivators demonstrated a stronger association with research activity compared to barriers, with the motivator “enhancing team credibility” being significantly associated with funding received. No significant association between self-reported research success and actual research activity was identified.

**Conclusions:**

Preliminary findings suggest that self-report measures of research success may not always correspond to actual research activity, and a combination of both these measures may be useful when planning RCB interventions. Variation in activity between teams and organisations should also be considered when tailoring RCB interventions. Reinforcing intrinsically motivating rewards of research may also be useful in promoting research participation for some teams.

## Background

The benefits of healthcare professionals participating in research are manifold. At a clinician level, such benefits may include increased use of evidence based practice, more positive perceptions and attitudes towards research [1], and higher job satisfaction [2]. At a team or organisational level, clinician participation in research may result in positive impacts on health-care performance including improvements in infrastructure and processes of patient care [3]. Societal level benefits are also reported, including the potential of more successful translation of research findings into clinical practice and greater societal impact of the research [4–6]. In light of such benefits, building the capacity of health care professionals to undertake research is considered a priority, and is of particular importance to the allied health workforce due to the relatively low evidence base for many allied health professionals’ (AHPs) interventions [7–9]. Indeed, initiatives targeting research capacity of the allied health workforce have recently been prioritised across different public healthcare organisations within Australia [10, 11] and internationally, for example in the United Kingdom [7, 12].

Research capacity building (RCB) has been defined as the process of development that aims to increase skills and abilities of individuals, teams or organisations in order to perform quality research [13, 14]. While interventions targeting RCB have historically been focussed on developing the skills and knowledge of individual clinicians [8], different studies of team-based RCB interventions have recently been reported with promising outcomes [13, 15, 16]. These team-based interventions employed a multi-strategy RCB approach comprising of research skills training, access to expert mentoring and quarantined time, and have resulted in increased research outputs and/or improvements in self-reported research capacity within single or multidisciplinary teams of varying sizes [13, 15, 16]. From a pragmatic perspective, Holden et al. [13] reported that targeting existing teams may also be a more efficient means of providing RCB strategies to more individuals whilst enhancing research culture within that team. Potentially increased efficiency of RCB strategies may be an important benefit, considering the current fiscal climate of Australian and other public healthcare settings.

There is still however much to be understood in regards to providing team-based RCB interventions within the allied health workforce. Authors emphasise the need to explore organisational context and developmental readiness of teams when implementing RCB, as these factors may potentially impact on a team’s research output [13, 15]. In order to devise effective RCB interventions, further insight into how AHPs are currently participating in research within their organisations is also indicated [17]. Greater understanding about research participation within teams and organisations may also elucidate how diversity can be accommodated for in respect to RCB rather than providing a “one size fits all’ approach [18]. This may be of particular consideration for the allied health workforce which is comprised of a number of diverse and disparate professions, making it unique to other workforces such as medicine and nursing [7].

Different measures exist which evaluate a team’s level of participation in research including their research output and performance. A recent systematic review revealed that the most common research performance indicators for studies of healthcare research were traditional academic indicators including number of publications, number of citations, impact factor and research funding [19]. Such measures are frequently used when reporting the research performance of health organisations in comparison to peers. There is however a need to extend these traditional measures of research output to also consider the clinical and societal impact of the research in terms of healthcare outcomes [19, 20]. Within the primary healthcare context, limited observational studies have reported the research outputs (or outcomes) of allied health teams. The majority of research to date investigating allied health research participation includes profession specific surveys [17, 21] or clinician’s self-report of their research activity and capacity, and associated enablers and barriers to conducting research [15, 22–24].

The largest study to date investigating research participation within the Australian allied health workforce used the self-reported research capacity and culture (RCC) tool to evaluate the research capacity at an individual, team and organisational level of 520 AHPs within the Victorian public health service [24]. The study found that participants who had a research lead (i.e., research position) working within their health service had higher ratings of team and organisational based research success. Higher team research success ratings were also associated with younger clinicians who had higher grade positions working within metropolitan health services. The team and organisational level findings from William’s et al.’s study validate previous research using the same tool across a group of AHPs [24] and individual professions including podiatry [25], dietetics [15] and psychology [26]. While such research adds to the evidence base of how different individuals across organisations are participating in research, further observational investigation into the research activity of specific allied health “teams” is still indicated. Indeed, the extent to which allied health teams are actually performing within their organisations in terms of their research activity and what factors facilitate or hinder their research participation remains largely unknown.

Pager et al. [23] conducted a survey on 85 AHPs from ten Australian healthcare teams describing the motivators, enablers, and barriers to building research capacity as an individual and within their team. Common barriers to undertaking research at an individual and team level were generally around extrinsic factors such as time and infrastructure. Individual motivators identified included intrinsic factors such as strong interest in research or opportunity to develop skills. Participants reported different motivators to undertake research for their teams, focussing on providing the best services and achieving optimal outcomes for patients. Although the study provided insight into the types of motivators and barriers AHPs encounter within their team, it is difficult to generalise findings to other settings as participants were from only one organisation. It is also unclear what impact these reported barriers and enablers were having on their team’s actual research activity. The relationship between motivators, barriers and actual research activity is a phenomenon beginning to be explored in the academic setting [27], yet remains largely untouched within the primary healthcare setting, particularly within allied health.

The positive impact of team-based RCB interventions are emerging in the literature [13, 15, 16], however in order to tailor and further develop such interventions, a clearer understanding of research participation within allied health teams is needed. Different self-report measures exist to evaluate a team’s research capacity, as well as associated barriers and motivators they have to participating in research. Research to date has however concentrated on collecting and analysing these measures at an individual rather than team level. It therefore remains unknown how such measures are associated with a team’s actual research activity (e.g., journal publications, funding received). In light of this, the present study aimed to identify the research activity, self-reported success, and motivations and barriers to undertaking research of eight AHP teams, and to explore whether any relationships exist between self-reported measures and actual research activity within each team.

## Methods

This study reports on the findings of an audit of research activity of eight AHP teams, and a prospective cross sectional survey of individuals within these same teams within one geographically located Australian health service. The survey was completed as a baseline as part of a broader RCB study. Ethical clearance was received for the study to be undertaken from the Gold Coast Hospital Human Research Ethics Committee (HREC/10/QGC/177).

### Audit of research activity

The first author collected research activity across all eight AHP uni-disciplinary teams (social work, psychology, physiotherapy, pharmacy, medical imaging, occupational therapy, nutrition and speech pathology) for a 20-month period (January 2013 to August 2014). Four outcomes were used to measure research activity within each team: (1) total number of journal publications authored by an AHP within the team, (2) amount (AUD$) of competitive research grant funding received (3) total number of conference presentations authored by an AHP within the team, and (4) total number of active ethically approved research projects which included an AHP team member as a principal investigator. The first three measures were chosen due to their frequent use in measuring research performance within healthcare [19, 28]. The fourth was chosen as it encompasses a number of research skills including literature review, research conceptualisation and protocol design, includes a stringent peer review process, and may capture the research activity of teams which exist outside of research funding [11].

The first author collected data for the audit by accessing local Human Research and Ethics Committee records, consulting with relevant employees from professions and teams (including professional team leaders), and accessing current registers and databases of research activity, funding and publications. Professional leads were invited to review the final data collected from the audit to ensure its accuracy.

### Survey

The Research Capacity and Culture (RCC) tool is a validated questionnaire which was developed to measure factors related to research capacity across three domains: individual, team and organisation [22]. As the present study was focussed on research activity of teams, only the team level data will be reported. The team level survey questions included 19 statements that respondents rated on a scale of 1–10 in terms of what they thought the success or skill level of their team was in relation to that particular statement. Responses of 1–3 indicated low success or skill, responses of 4–7 indicated average success or skill and scores of 8–10 indicated high success or skill. The RCC tool also included a section whereby the respondent identified whether or not they had experienced a list of different motivators and barriers to undertaking research as an individual or within a team. To address the study aims, only team level motivators and barriers were used for analysis. For this part of the questionnaire, respondents were required to indicate which, of a list of up to 18 motivators and 19 barriers, were relevant to their team when undertaking research, with participants being able to indicate as many items as they considered relevant to their team. The survey included additional questions related to basic demographics including years of experience and whether they had completed a research post-graduate qualification. The survey was distributed via a secure web-based survey platform and the survey link was sent to all AHP heads and/or team leaders to forward to their staff (totalling approximately 600 employees). A number of reminders (i.e., through organisational bulletins and team meetings) were given during the survey period, with the survey remaining open for approximately 4 weeks within August 2014.

### Data analyses

Survey results of the RCC tool, and research activity data were analysed descriptively with mean scores within each AHP team being reported by the first author. Due to the non-normal distribution of the data, Kendell’s correlation coefficient was used to determine whether any association existed between average self-reported measures of success at research from the RCC tool with research activity (i.e., journal publication, funding and number of approved research project ratios), as well as whether a relationship existed between the percentage of specific barriers and motivators reported within each team with research activity. Multiple comparison adjustments using the Bonferroni method were made to answer each research question, with a *p* value < 0.002 required to be considered statistically significant.

Due to variation in the number of employees within each professional team (see Table 1), research activity measures were divided by the total number of full time equivalent (FTE) AHPs in each team. Data was therefore expressed as the number of publications per FTE staff member, number of ethically approved projects per FTE staff member, and amount of funding ($) per FTE staff member for analyses. Due to the high correlation between journal publications and conference presentations, the latter measure was not included in the correlation analyses. Within the RCC tool, only the top six motivators and barriers that participants’ indicated as relevant for their team (i.e., when all data was combined) were included in the correlational analyses. Teams were labelled numerically in random order rather than by profession to respect the team’s anonymity.

**Table 1:** Research activity according to team

Allied health professional team	Total no of staff (Full time equivalent)	Journal publications 2013–2014	Conference presentations 2013–2014	Total grant funding received 2013–2014	No. ethically approved projects
Team 1	85	0	3	<$10,000	6
Team 2	95	1	3	<$10,000	2
Team 3	82	0	4	$10,000 > $50,000	5
Team 4	70	0	0	$10,000 > $50,000	3
Team 5	68	0	0	<$10,000	0
Team 6	91	2	10	$50,000 > $100,000	4
Team 7	37	3	4	>$100,000	8
Team 8	40	4	7	$50,000 > $100,000	4
Average	71	1.25	3.87	$10,000 > $50,000	4

## Results

### Audit of research activity

Teams were comprised of an average of 71 full time equivalent AHPs (range = 37–95) as shown in Table 1. The number of journal publications and conference presentations, total grant funding and number of research projects ethically approved per team from January 2013 to August 2014 are shown in Table 1. The amount of grant funding is presented categorically after being originally calculated from raw values. The audit revealed that all but one team were undertaking at least two ethically approved research projects. Four and six out of the eight teams had produced journal publications and conference presentations respectively, and five of the eight teams had received grant funding over $10,000 during the 20-month audit period.

### Survey results

A total of 158 allied health staff visited the RCC survey. Five people chose not to complete the survey after visiting the website, with the remaining 153 staff providing consent to participate in the survey. As not all questions were mandatory, a number of participants did not complete all survey questions. A total of 95 (approximately 17% of total AHPs) complete surveys from participants were available for analyses across the eight teams (see Table 2). Mean response rate for teams was 18% (range = 12–24%), with respondents generally female and spread across years of experience, however there was generally less representation from newer graduates (i.e., having less than 2 years of experience).

**Table 2:** Demographics of allied health professional teams and respondents in survey

Allied health professional team	No. participants in survey	Response rate in team (%)	% Years of experience in profession					% staff with postgraduate qualifications in survey	% female
			<2 years	2–5 years	6–10 years	11–15 years	>16 years		
Team 1	7	12%	29%	29%	29%	15%		100%	71%
Team 2	15	16%	7%	7%	40%	13%	33%	46%	`93%
Team 3	14	17%		14%	29%	21%	36%	71%	36%
Team 4	15	21%	7%	13%	40%	7%	33%	73%	60%
Team 5	10	15%			40% ^a^		50%	0	50%
Team 6	15	16%		27%	20%	27%	27%	0	93%
Team 7	9	24%	22%	22%	33%	22%	11%	0	100%
Team 8	10	25%		40%	40%	10%	10%	20%	100%
Average		18%	8%	19%	34%	14%	25%	39%	73%

^a^ One respondent did not respond to this question

Average responses to the RCC tool for each of the team level items are found in Table 3, with shaded numbers indicating teams’ mean scores that were reported as low success (i.e., score <4). The respondents reported their teams as having average success for the majority of items apart from Team 5 which rated 16 out of 19 of the items as having low success. Certain items were however rated as low success by most teams including having adequate resources for research training, funds and administrative support for research, mechanisms to monitor research quality, incentives for mentoring and software availability. Apart from three items in Team 1, there was generally a lack of scores in the high success range (i.e., 8–10).

**Table 3:**
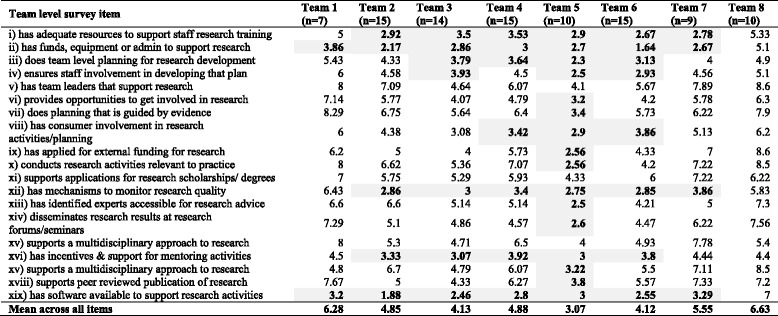
Mean score for team level RCC survey items across AHP teams

Note shaded areas represent low average success level rated for this item

Table 4 presents the frequency of reported barriers and motivators to undertaking research within a team averaged across all 95 participants. The most frequently reported barrier to research was lack of time for research, followed by other work roles that took priority and the lack of suitable backfill. The most frequently reported motivators were to develop skills and increase job satisfaction, followed by career advancement. Respondents identified motivators to research generally less frequently than barriers.

**Table 4:** Reported frequency of barriers and motivators across entire sample

Barrier to research within team	% of response across *n* = 95	Motivator to research being conducted within team	% of response across *n* = 95
Lack of time for research	90.53	To develop skills	63.16
Other work roles take priority	77.89	Increased job satisfaction	61.05
Lack of suitable backfill	66.32	Career advancement	55.79
Lack of funds for research	57.89	Increased credibility	45.26
Staff shortages	57.89	Problem identified that needs changing	44.21
Lack of administrative support	55.79	Links to universities	43.16
Lack of skills for research	54.74	Desire to prove a theory/hunch	34.74
Lack of software for research	50.53	To keep the brain stimulated	32.63
Different experience levels of team members	46.32	Colleagues doing research	28.42
Lack of a co-ordinated approach to research	34.74	Mentors available to supervise	28.42
Lack access to equipment for research	33.68	Research encouraged by managers	28.42
Desire for work/life balance	31.58	Dedicated time for research	25.26
Intimidated by research language	28.42	Team building	24.21
Lack of support from management	20.00	Study or research scholarships available	22.11
Other personal commitments	18.95	Opportunities to participate at own level	22.11
Intimidated by fear of getting it wrong	14.74	Grant funds	21.05
Isolation	13.68	Forms part of Post Graduate study	20.00
Not interested in research	12.63	Research written into role description	12.63
Lack of library/internet access	5.26		

### Correlation with research activity

The mean survey scores for self-reported research success at a team level did not significantly correlate with any of the research activity measures. Further exploratory analyses of each of the 19 items of the RCC tool also revealed no significant correlation with research activity measures. The six most frequently reported barriers and motivators across all respondents are presented in Tables 5 and 6, together with correlations with research activity measures. No significant correlations were found between any of the top six barriers and research activity (see Table 5). A significant positive correlation was found between the frequency of reporting “increased credibility” as a motivator and the amount of research funding received within teams (see Table 6). The association between this motivator and amount of journal publications and ethically approved research also approached statistical significance. Other motivators demonstrating an association with at least one of the research activity measures that approached statistical significance included job satisfaction, skills development and identification of a problem that required changing.

**Table 5:** Frequency of reported team barriers to undertaking research and correlation with research activity

Team barrier	% frequency of participants reporting team barrier								Correlation analyses with research activity					
	Team 1	Team 2	Team 3	Team 4	Team 5	Team 6	Team 7	Team 8						
	(*n* = 7)	(*n* = 15)	(*n* = 14)	(*n* = 15)	(*n* = 10)	(*n* = 15)	(*n* = 9)	(*n* = 10)	Journal ratio		Funding ratio		No. projects	
									r_τ_ =	p=	r_τ_ =	p=	r_τ_ =	p=
Lack of time for research	71	93	100	93	90	93	89	80	-.372	.364	-.149	.725	-.184	.663
Clinical backfill	57	67	86	53	80	60	55	70	-.209	.619	-.278	.504	-.337	.414
Other work roles take priority	100	80	93	67	90	67	78	60	-.524	.182	-.482	.227	-.129	.761
Lack of funds	86	47	64	73	30	53	67	50	-.034	.937	-.067	.875	-.405	.319
Lack of admin support	57	67	64	40	50	73	33	50	-.412	.310	-.587	.126	.-562	.147
Staff shortage	43	53	57	47	80	60	33	90	.198	.168	-.057	.893	-.422	.298

**Table 6:** Frequency of reported team motivators and correlation with research activity

Team motivator	Frequency of reported motivator (%) from each professional team								Correlation analyses with research activity					
	Team 1	Team 2	Team 3	Team 4	Team 5	Team 6	Team 7	Team 8						
	(*n* = 7)	(*n* = 15)	(*n* = 14)	(*n* = 15)	(*n* = 10)	(*n* = 15)	(*n* = 9)	(*n* = 10)	Journal ratio		Funding ratio		No. projects	
									r_τ =_	p=	r_τ =_	p=	r_τ =_	p=
To develop skills	43	60	64	53	53	67	78	100	.871	.005*	.788	.020*	.573	.137
Career advancement	43	53	50	67	67	47	67	70	.667	.071	.727	.041	.506	.201
Increased job satisfaction	57	33	50	73	73	47	89	100	.792	.019	.804	.016*	.639	.088
Links to universities	57	47	50	40	40	40	56	40	.216	.607	.277	.507	.612	.107*
Problem needs changing	43	33	57	33	40	13	78	80	.759	.029*	.774	.024*	.777	.023*
Increase credibility	43	33	43	40	40	53	67	60	.856	.007*	.907	.002**	.862	.006*

* = significant at *p* = <.05, ** significant after multiple comparison adjustment

## Discussion

The present study described the research activity, self-reported success level, motivations and barriers of eight AHP teams in undertaking research and explored whether any relationships exist between the self-reported measures and audited research activity within each team. The study revealed variation in research activity between teams, however the majority of teams were undertaking some form of ethically approved research and had disseminated their research. All teams, apart from one, reported average success at undertaking various aspects of research. No significant correlations were found between self-reports of research success and team barriers to undertaking research with research activity. Motivating factors were found to have a stronger association with research activity, with enhancing team credibility having a significant correlation with funding received.

The most consistent form of research activity across teams was undertaking ethically approved research projects and conference presentations. The finding that the majority of teams were presenting research despite any dedicated research staff within their team is consistent with a recent study of physiotherapy departments across Australia [21]. Although survey findings of our study revealed that respondents rated their teams as being average at “supporting peer reviewed publication of research”, the mean number of publications from AHP teams in the present study was considerably lower than figures reported by other Australian allied health teams [21]. For instance, Skinner et al. [21] reported a median number of 6.5 articles across 24 months per Physiotherapy team. While Skinner et al., acknowledge a potential response bias in their study, publication rates are still considerably higher compared to the present study which revealed an average of one publication per AHP team across a comparable timeframe [21]. The teams’ low publication rate in the present study may be consistent with other literature which recognises that publication in peer-reviewed journals is often considered a difficult task for clinicians in allied health as well as medicine and nursing [15, 17, 24, 29–32]. The paucity of publications that teams produced could potentially be related to the reported lack of time, which was the most frequently reported barrier to undertaking research. Although this barrier was not found to be significantly associated with journal publication ratio or other research activity outcomes, the time needed to prepare a grant application, write an ethics application or prepare a conference presentation is considerably less compared to writing a quality manuscript suitable for publication and the review process that follows.

Although no significant association was found between the self-reported measures of research success from the RCC tool and actual research activity, some patterns could be identified between the two measures. Findings appeared to show that teams within the organisation with either high or low research output were more accurate in self-reporting their research success, while teams performing in the middle were less consistent. For example, the audit revealed Team 5 to be the only team not participating in any research activity, and this team also rated themselves as having the lowest research success. Teams 2 and 8 yielded the most research funding and publications amongst the audited teams and both rated themselves on the high average end (averaging >5.5 across items) in terms of research success. Team 1, which was in the middle in terms of research activity compared to other teams (i.e., had six ethically approved projects but did not have any funding or publications), however rated themselves as high average on the RCC tool for a number of items. One could argue that social desirability bias, which suggests that participants rate themselves more positively than their actual performance to produce a more favourable image [33], may potentially account for some of the discrepancies between the self-report of a team’s research success and the same team’s actual research activity in the present study.

Results of the research activity audit also highlight that at a given time point, considerable variation between allied health teams within the same organisation exist. This finding potentially challenges the notion that AHPs can just be categorised together in terms of their RCB needs [7], and suggests that AHP teams may differ in respect to what RCB targets they may require. In the same way, findings revealed that teams may also differ in their motivations for research. Indeed, a number of frequently reported team motivators identified in the present study including career advancement, increased credibility and job satisfaction were only reported as minor themes in another Queensland Health study of AHPs [23]. This suggests that motivating factors towards participating in research may also vary from team to team as well as across organisations.

A number of the motivators in the present study also showed trends of a positive relationship with measures of research activity, with “increased credibility” being most strongly associated (and statistically significant). This motivating factor was also reported in a study of Australian podiatrists, and was suggested to be potentially more applicable to smaller professions as they may feel that they need to advocate for themselves more in order to demonstrate their credibility [25]. Interestingly, the two teams with the highest overall research activity (i.e., Teams 2 and 8) were also the smallest. It could be speculated that the desire to want to increase team credibility allowed these teams to push past barriers to produce more research activity (i.e., apply for research funding) compared to other larger teams. It should also be noted that both of these teams also had professional directors who were actively involved in research projects. This manager level role modelling and support of research may therefore also have assisted the team’s productivity despite their small size.

An interesting finding of the present study was that motivating factors were more strongly associated with research activity compared to barriers. This outcome is consistent with behaviour theory. According to Herzberg’s two-factor theory, eliminating negative factors or barriers does not always result in motivating employees to achieve a goal [34]. In other words, regardless of the presence or absence of barriers, a team may still not be motivated to engage in research. To motivate employees towards a goal, intrinsic rewards such as opportunities for personal development or growth, achievement or recognition are required [34]. According to this theory, it could be postulated that individuals within the teams that were motivated by the intrinsic rewards of research (i.e., leading to enhanced team credibility, skill development, job satisfaction, solving an identified problem) therefore tried harder and were more persistent at producing research outputs despite experiencing similar organisational barriers to other teams. Although not in the health field, a previous study of academics also found a positive correlation between intrinsic motivation and research productivity and found scientists who took a stronger interest in their research were more productive [27]. In the same way, the present findings suggest that AHP teams that are generally more interested in research, driven by their internal motivations towards research, may be more research productive.

### Limitations

Due to certain limitations of the present research, results should be interpreted conservatively. Participants were all from a single geographical location and therefore results may not generalise to the wider allied health workforce. Due to low participant response, only a cross section of participants from within each team completed the RCC tool with a disproportionate representation from more senior staff. This may have resulted in some potential response bias. While a number of efforts were used to increase respondents, it is likely that clinicians had little incentive to prioritise completing the survey within their busy clinical caseloads. Further incentives (i.e., prize give away), extending the survey deadline, and local “champions” to encourage completion of the survey may have further assisted. For the purpose of this research only the research activity of the professional team was measured, however it is also acknowledged that AHPs frequently work in other smaller multi-disciplinary teams within their workplace (i.e., service based units). In the present study, teams were categorised into their professions because the majority of clinicians within the organisation received their professional, operational and strategic oversight from within these professional teams (i.e., attending departmental meetings and professional development training together). Although multi-disciplinary teams were not observed within the present study, collaborative research teams across professions should be considered in ongoing RCB research, as they may be potentially more beneficial [35].

### Recommendations for future team-based RCB

Current findings highlight some potential considerations for team-based RCB interventions and research. While tailored interventions using a “whole systems” approach has been recommended when it comes to allied health RCB [24], present findings reflect that such initiatives undertaken within an organisation may need to target specific professions or teams separately according to their profile of activity and research ability. Results also suggest that self-report measures of research success or skill should be interpreted together with the team’s current levels of research activity to establish an overall understanding of a team’s research capacity. This may be a particularly important consideration for dedicated research positions employed within healthcare organisations, which are becoming more prevalent within Australian public healthcare settings to promote research capacity [10, 24]. The use of research registers to capture and monitor research activity within teams may also be beneficial [36], with such information being used to assist in establishing, tailoring and evaluating team-based RCB interventions. For example, teams that are currently not undertaking any active research projects are unlikely to benefit from interventions targeting manuscript writing. Further support or training to individuals in these teams may be needed regarding formulating research questions and preparing a research protocol or ethics application. Then, once a project is successfully commenced within a team, clinicians within that team may be able to role model learnt skills to other clinicians, thereby creating a “snowball effect” or accumulative “grow your own” approach to RCB [33]. Indeed, such vicarious modelling can be effective in building staff’s self-efficacy [37]. Moreover, at a team level the success in completing research may in turn increase an individual’s belief that their team can succeed thereby increasing “team efficacy” [19].

The finding that motivating factors were more strongly associated with research activity suggests that interventions aiming to increase research activity within AHP teams should consider evoking intrinsic motivations, including how the team can use research to promote their team’s reputation, address meaningful clinical problems, advance their career and increase their job satisfaction. Doing so may help to maintain the intrinsic motivation needed to persevere in research activity within clinically demanding environments. Further research across different organisations exploring the link between self-reported barriers and motivators and actual research activity may also be indicated to further substantiate this notion. One potential avenue of enquiry may be investigating whether targeting intrinsic motivations of a team results in enhanced research activity compared to addressing team barriers alone and/or a combination of both barriers and motivators.

## Conclusions

While previous studies have collated research activity from specific allied health professions, this is the first observational study, to our knowledge, that has reported current research activity across existing AHP teams within an Australian public healthcare organisation. The research adds to the existing knowledge base by providing an example of the current level of research activity different AHP teams undertake (within an outer metropolitan health organisation) prior to any formal RCB intervention and how this activity is associated with self-report measures. Findings revealed variable yet generally low research activity across AHP teams and limited association between self-reported success and barriers to research and actual research activity. In contrast, a number of motivating factors had a strong association with research activity.

The present findings also reveal some key messages, which may help inform future RCB interventions and research in the area. Firstly, when evaluating a team’s research capacity, self-report measures should potentially be considered in combination with actual research activity and outputs (i.e., captured on a research register). This may provide a more holistic picture of the team or profession’s current level of research participation. Secondly, variation between professions, teams and organisations in terms of research activity undertaken and self-reported research capacity should be considered when tailoring RCB interventions and a one size fits all approach is unlikely to be effective across all teams within an organisation. Thirdly, when attempting to promote research participation and activity amongst teams, there should be a greater focus on enhancing motivating factors and intrinsic rewards of research such as increased credibility and developing skills, and ability to address meaningful clinical problems.

## Abbreviations

AHP: Allied health professional
FTE: Full time equivalent
RCB: Research capacity building
RCC: Research capacity and culture
